# Online Graduation of Doctors During the COVID-19 Pandemic

**DOI:** 10.15694/mep.2020.000122.1

**Published:** 2020-06-18

**Authors:** Andrew Blythe, Imogen Jones, Natasha Chakraborty

**Affiliations:** 1 University of Bristol

**Keywords:** COVID-19, Teaching, Medical School, Lockdown, Online Graduation, Qualification, Medical Students, Doctors

## Abstract

This article was migrated. The article was marked as recommended.

In March 2020, the United Kingdom declared a nationwide lockdown, a public health intervention in response to the COVID-19 pandemic. In addition, the increasing pressures on the country’s publicly funded healthcare system, the National Health Service, required the early graduation of final year medical students so that they could join the workforce. Bristol Medical School responded to this health crisis by graduating 226 final year students. Since social distancing policies resulted in the prohibition of social gatherings, university graduation ceremonies were cancelled. The medical school felt it important to mark the students’ qualification as these young doctors were to begin their careers amidst an unprecedented global health emergency. An online graduation ceremony was held on the video conferencing platform Zoom. This was attended by university staff and students from their homes across the UK and elsewhere in the world. Students commemorated their qualification by submitting photographs of themselves celebrating in homemade graduation robes and mortar boards which were included in the online event. The ceremony was a memorable occasion and created a sense of community in a time of social isolation. This novel situation gave rise to a unique celebration attracting media coverage and was reported on national TV and radio news bulletins along with newspapers. The structure for the online graduation ceremony outlined in this paper may be replicated for other graduation ceremonies or celebrations affected by the COVID-19 lockdown.

## Introduction

A graduation ceremony marks the successful completion of years of hard work and for medical students it also marks the transition from student to doctor. The ceremony is a time when students and staff can celebrate in the presence of those who have supported them over the longest time: their close family. The Coronavirus disease (COVID-19) pandemic has simultaneously created a need for the early graduation of final year medical students whilst denying them the opportunity to do this in person. On Friday 3 April 2020 Bristol Medical School responded to this crisis by graduating 226 final year students in an online ceremony. Since then many other medical schools have held virtual graduation ceremonies or are planning to do so. In this paper we describe how we organised this event and what we learned in the process of doing so.

In the UK most graduation ceremonies take place during the summer so that new graduates can begin work in the National Health Service (NHS) in August. On 24 March 2020 the United Kingdom Secretary of State for Health announced that graduation should be brought forward, enabling the newly qualified doctors to bolster the NHS workforce. By good fortune Bristol Medical School was well placed to do this because our students had sat all their final exams and were due to complete their last core clinical placement on 27 March 2020. After this time students are usually on their elective placement. However, the elective period was abandoned and exam boards were brought forward to 31 March 2020 to confirm that our students had qualified. Meanwhile all university graduation ceremonies were cancelled due to the public health policy of social distancing and banning of mass gatherings.

We felt we needed to mark our students’ qualification in some way. They were disappointed that they had lost their elective, anxious about starting work at a time of national crisis and upset they would not be able to see each other to celebrate. Recently published literature suggests that the cancellation of university operations and the uncertainty it causes, negatively impacts the mental wellbeing of students and staff alike ( Kafka, 2020; Sahu, 2020). This might be felt by medical students who are joining the workforce at such a difficult time ( Al-Rabiaah *et al.*, 2020). Therefore, we decided to attempt an online graduation ceremony to boost morale and celebrate the hard work of our students. We set ourselves a very short deadline: 3 April 2020, which left just two weeks to organise and orchestrate the virtual ceremony.

## What did we do?

We used Zoom Pro, a video conferencing platform that allows unlimited participants with no restriction on the length of the meeting. We had tried this once already with our final year students with a Zoom meeting hosted by the Dean of the Medical School on 25 ^th^ March to discuss the changes to their curriculum; it had worked well.

Conscious of the newly described phenomenon of Zoom-bombing (in which uninvited guests gate-crash and disrupt meetings) we sent the link and password for the meeting to 66 members of staff in addition to the 226 new graduates. We told all invitees that they would have to sign onto Zoom with their full name so that we could check their identity. We had two members of IT staff monitoring the event. Limiting the numbers of invitees also made the event more manageable.

All participants, including staff, joined the Zoom from their own home and were asked to turn their video on if they wanted to be seen. All microphones except for those of the speakers were turned off.

We kept the ceremony simple but adhered roughly to the format of a normal graduation ceremony. The event started with a welcome from the Dean of the Faculty of Health Sciences followed by a brief introduction of each of the key members of the University Senior Management Team who were in “attendance”. Next, the President and Vice-Chancellor of the University gave an address to the students and staff. Then the Dean read the names of all the newly qualified doctors in alphabetical order. Normally each student would go on stage to be greeted by the Vice-Chancellor and be applauded by the audience. For our online graduation the Dean read the names in batches of 20 and then paused whilst all the microphones were turned on so that people could applaud or cheer. At the end of this roll call the Director of the MB ChB programme gave an address before closing the event and turning on all the microphones for one final cheer.

**Figure 1.**
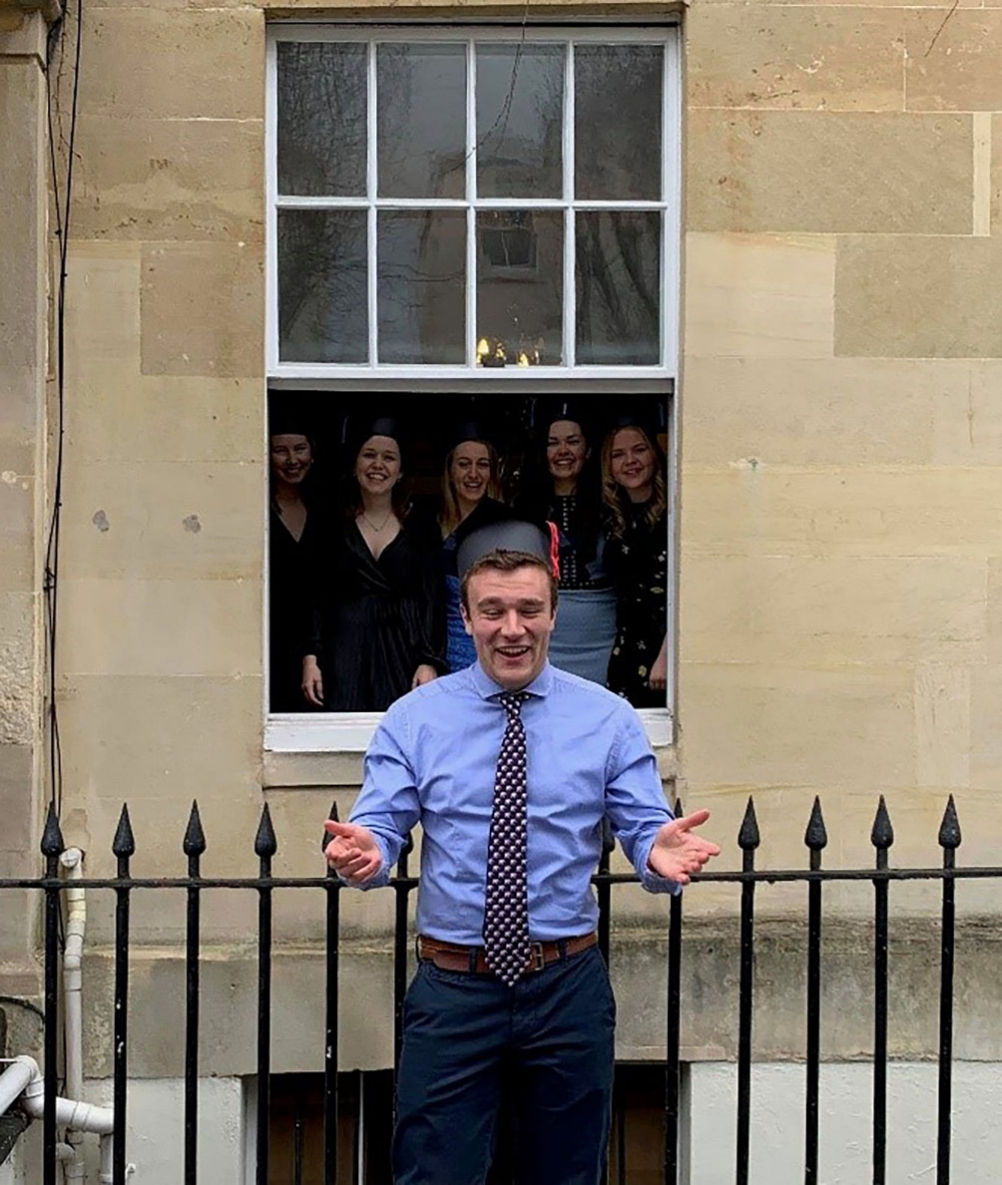


**Figure 2.**
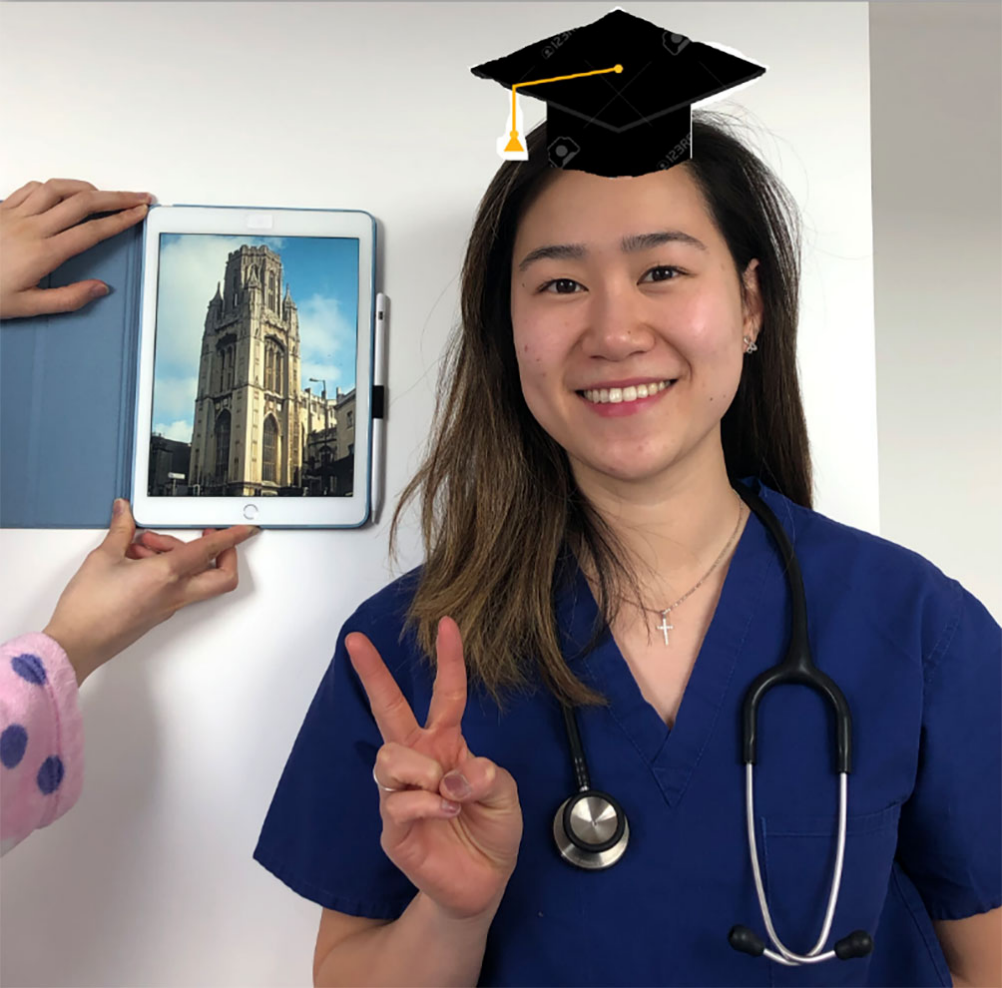


**Figure 3.**
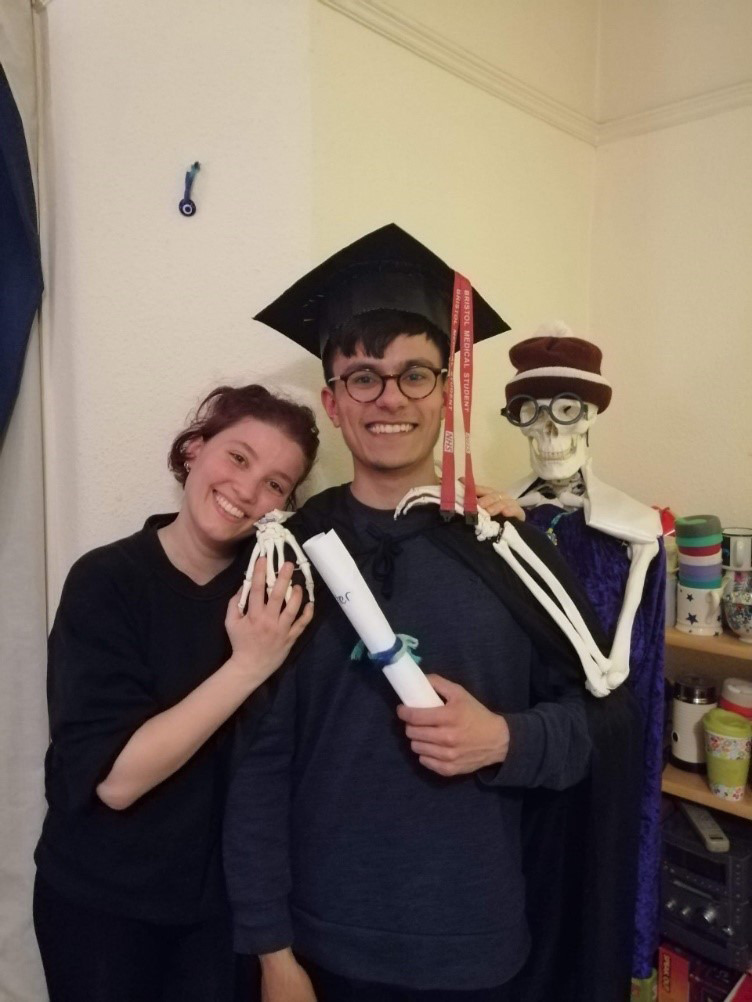


**Figure 4.**
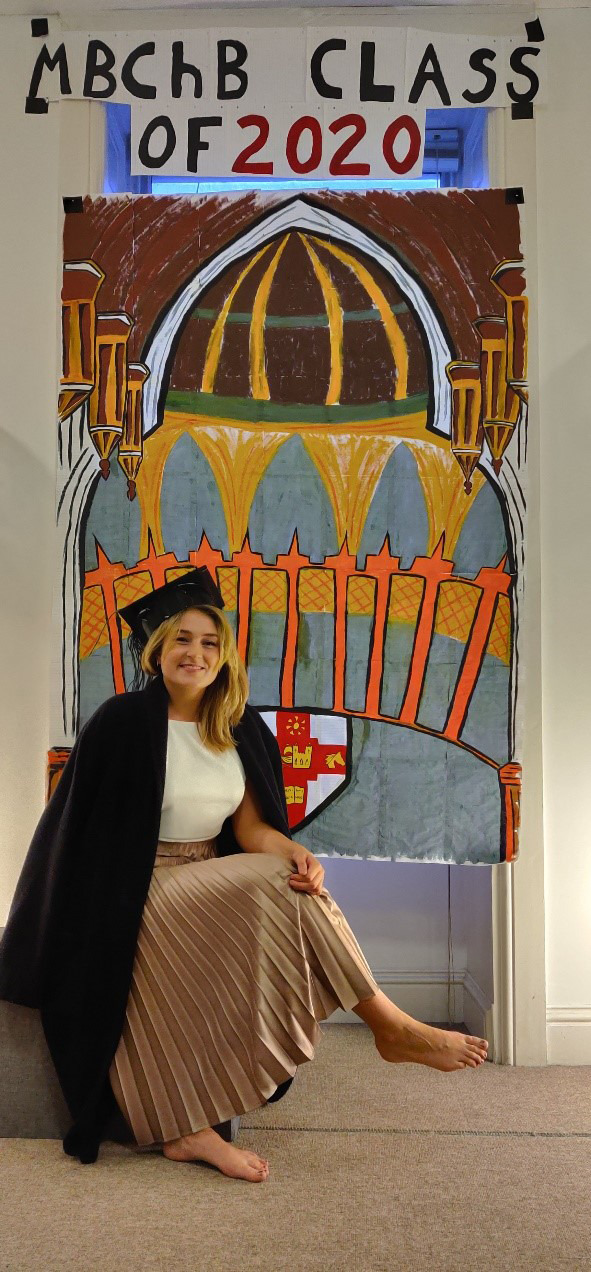


The students were keen to help with the planning of this event and under the leadership of one representative, submitted photos of themselves, before the event. In these photos many students dressed up in homemade graduation robes and mortar boards. A member of staff collated these photos in a Power Point presentation that was screened at the start of the ceremony. Participants were invited to log onto Zoom up to 15 minutes before the ceremony started and could see these pictures on a continuous loop as soon as they logged-on. The pictures were shown again at the end of the ceremony. Examples of the photos are shown in Figures 1 to 4.

To conceal their true location (study/living room) the three key speakers at the event used a common background photo of the interior of the Great Hall of the Wills Memorial Building, where all graduation ceremonies normally take place at the University of Bristol.

Like many platforms for online meetings, Zoom Pro has a chat facility in which participants can post comments during the meeting. We allowed all participants to use this chat facility throughout the meeting. The comments appeared in real time on the right-hand side of everyone’s screen and were not edited in any way.

In our normal graduation ceremony, the newly graduated doctors recite the Bristol Promise, a modern version of the Hippocratic Oath written by students themselves and the University’s first Professor of Medical Ethics and Law. We did not ask the doctors in the online graduation ceremony to do this because we were unsure of how it would sound when transmitted through 226 individual microphones.

We created a simple programme for the event and circulated this in advance of the ceremony for participants to print off if they wanted to do so. This programme consisted of the order of ceremonies, a list of the new graduates’ names, a message from the academic lead of the final year of the programme and the Bristol Promise.

## What worked well?

We had been fearful that the IT would let us down, but we are pleased to say this was not the case. There were two minor snags: 1) we had wanted to highlight the senior members of university staff when they were introduced by the Dean, but it was difficult to do this quickly when their images were located amongst so many other participants and 2) the software initially mistook loud applause as background noise and tried to edit it out.

The ceremony was surprisingly interactive and inclusive. All participants chose to turn on their video so that they could be seen; they waved and put up messages. Students who were living at home could be seen with their family around them. Normally each student is only allowed two guests, but in this online ceremony there was no limit to the number of guests providing they were all living together and able to share the same screen. More staff attended the event too. The chat facility allowed everyone to communicate with each other throughout the ceremony. In a normal graduation ceremony, participants are restricted to whispering to their neighbors in the hall and cheering or clapping as the new graduates go on stage. In the online ceremony everyone had the ability to communicate to everyone else.

The ceremony blended formality with informality. The two addresses and roll call mirrored the formality of normal graduation ceremony. In contrast, the montage of students’ photos in homemade outfits and the chat facility made the event feel more like a family gathering. Students used the chat facility to congratulate each other and thank staff. Staff used the chat facility to congratulate students and comment on the ceremony in general.

Most significantly the event was a very moving occasion. Immediately after the ceremony our Pro Vice-Chancellor for Education wrote:

“That was easily the most moving graduation ceremony I have ever attended... the chat was extraordinary -funny, poignant and inspiring”.

The students and some parents wrote to express a similar sentiment. One student’s parents wrote:

“As you can imagine after 6 years at medical school, we were so looking forward to seeing our daughter (X) graduate and had resigned ourselves that nothing could be done during these Coronavirus restrictions. We were so delighted that the ingenuity of the Zoom graduation and seeing all the fantastic photos of our newest doctors was so uplifting”.

The event was recorded and a link to the recording was distributed all new graduates within 24 hours so that they could view it again and share the event with their family and friends.

The event attracted considerable interest from the local and national press: radio, television (TV) and newspaper. This made the event even more special for the newly graduated doctors. Their graduation does not normally get reported on the evening TV news.

## What would we do differently if we were running the event again?

### Event logistics

The event lacked music. At a normal graduation ceremony, the arrival and departure of the academic staff is marked by loud organ music which echoes through the Great Hall, adding to the grandness of the event. In contrast, when participants logged onto the Zoom Pro event, they watched the photos in silence.

During the roll call one student put a comment on the chat board to say that her name had been missed. This was “rectified” in the second address. On viewing the recording however, it turned out her name had been read out by the Dean, but she had been so busy cheering her peers that she had missed her name being read out. Perhaps reading names more slowly will avoid such misunderstandings in future virtual celebrations.

### Media coverage of the event

Our celebration was reported by a number of media outlets including the TV evening news along with a handful of radio stations and newspapers. These media reports highlighted the emotion generated by the extraordinary context of the ceremony and the work facing the newly graduated doctors.

It was brought to our attention by a new graduate that much of the media coverage of our online graduation displayed an unconscious racial bias. The majority of students approached by the media and pictured in news articles were Caucasian; this did not give an accurate representation of our new graduates and doctors. 23% of our new graduates are from a Black, Asian and minority ethnic (BAME) background. 42% of doctors in the UK are of a BAME background, ( Dunne, 2017) yet the fair portrayal of BAME people has long been an issue in media. ( Campion and Taylor, 2017) This issue felt pertinent to address, especially in the current climate, with the pandemic highlighting stark inequities of disease. COVID-19 has been attributable to a disproportionate number of deaths in the BAME community (Intensive Care National Audit and Research Centre, 2020).

Bristol Medical School has a strong emphasis on widening participation reflected through initiatives such as our MB ChB Gateway to Medicine programme, where we aim to end discrimination and close the BAME student attainment gap in higher education. Taking steps to ensure fair portrayal of BAME students by the media is one thing we would do differently next time. This issue was shared with the University Press Team to help them develop their policies for the future as well.

## Conclusion

The online graduation ceremony served its purpose well. It was a memorable and moving way of marking a very significant event in the lives of 226 new doctors and their families. It was the first online graduation ceremony held by the University of Bristol and possibly the first in the UK. This format may be used again for other graduation ceremonies that have been affected by the COVID-19 lockdown.

## Take Home Messages

• An online graduation ceremony is an uplifting alternative when university graduation ceremonies are cancelled as a result of national lockdown.
• Zoom and other video conferencing programmes are suitable platforms to host large online events such as a graduation ceremony.
• It is vital to take steps to ensure the fair portrayal of BAME students in the media coverage of university events.

## Notes On Contributors

**Dr Andrew Blythe**, MRCGP, DRCOG, DCH, is the Director of the MB ChB Programme at Bristol Medical School, University of Bristol, Bristol, United Kingdom. He is also a General Medical Practitioner (GP) at Bridge View Medical in Bristol.

**Dr Imogen Jones**, MB ChB, BSc, is a new graduate from the University of Bristol, Bristol, United Kingdom.

**Dr Natasha Chakraborty**, MB ChB, is a new graduate from the University of Bristol, Bristol, United Kingdom.

## Appendices

*Glossary*

*Elective*

A medical elective is an educational placement in a clinical setting that is chosen and organised by individual medical students. These placements may be in the UK or overseas ( General Medical Council, 2009).

*Social distancing*

Social distancing is a public health measure implemented to minimise social interaction between people (GOV.UK, 2020 ^a^).

*Lockdown*

A lockdown is the government enforced closure of schools, workplaces and non-essential services, in order to maintain effective social distancing in a population (GOV.UK, 2020 ^b^).

## Declarations

The author has declared that there are no conflicts of interest.

## Ethics Statement

This paper describes how we organised an online graduation ceremony. The students were not asked to complete a questionnaire and were not the subject of any potentially harmful intervention.

## External Funding

This article has not had any External Funding
