# Supplementary material for: Online Graduation of Doctors During the COVID-19 Pandemic
Source: MedEdPublish (2016). 2020 Jun 18;9:122. [Version 1] doi: 10.15694/mep.2020.000122.1 (PMC10702667; doi:10.15694/mep.2020.000122.1)
Supplement: Peer review history

# Reviewer report: Reviewer response for version 1

**DOI:** 10.21956/mep.20121.r31038

**Published:** 2020-10-21

**Authors:** Ken Masters

**Affiliations:** 1 Sultan Qaboos University

This review has been migrated. The reviewer awarded 4 stars out of 5 A really great paper detailing the online graduation ceremony of doctors during the COVID-19 pandemic. This part of obtaining one’s qualification is an important component of social recognition, and it is, indeed, a pity that the pandemic has denied the students the opportunity to attend in person. The university has done well to react positively to this, and appears to have really pulled it off successfully. I am sure that, by their writing this paper, the authors have inspired others to perform similar ceremonies, and the document also stands as a record for the future. Far from having had a disadvantage, these students will be able to look back with joy at (and bore their grandchildren with the story of :-) their graduation.In addition, my hope is that, even when graduation ceremonies return to being face-to-face events, a paper like this can serve as a reminder that a hyflex approach is possible. So, for those who cannot attend in person, they can attend online, using what will then be fairly commonplace technology. Well done all round.

Reviewer Expertise:

NA

No decision status is available

# Reviewer report: Reviewer response for version 1

**DOI:** 10.21956/mep.20121.r31040

**Published:** 2020-07-27

**Authors:** Gregory Neal-Smith

**Affiliations:** 1 Royal Berkshire Hospital

This review has been migrated. The reviewer awarded 5 stars out of 5 This is an excellent write up of a novel solution for dealing with the coronavirus pandemic. I can imagine the students found this experience hugely rewarding, given their hard work over the last 6 years. It also addresses relevant issues about BAME representation that is important to discuss in the current climate. It is nicely balanced with areas of focus for future development, which is reasonable considering a new event like this is likely to have a few teething problems.I'd be interested to see some quantitative feedback from the students about the experience, and also whether this idea is being developed by Bristol University for other events? If the feedback is good then I wonder if this is something the the BMA/GMC could develop for other medical schools?Great article to read and congratulations on the success of the event!

Reviewer Expertise:

NA

No decision status is available

# Reviewer report: Reviewer response for version 1

**DOI:** 10.21956/mep.20121.r31039

**Published:** 2020-06-19

**Authors:** P Ravi Shankar

**Affiliations:** 1 American International Medical University

This review has been migrated. The reviewer awarded 4 stars out of 5 This is a very readable and lucidly written account of graduation during a time of lock down and university closures. The authors have described the entire process very well. They also highlight that most of the media focus was on the Caucasian students. They mention both the advantages and the drawbacks of the online ceremony.An online graduation is an event which can serve the purpose of recognizing student achievement and bringing people together during the pandemic. However, after attending many online meetings, webinars and courses I must admit that online does not substitute ‘adequately’ for a physical ceremony and interaction. Students must have missed many of the ceremonies and the ambiance of the hall where the ceremonies would have been held. I am sure most would have missed the graduation dinner. I am not sure whether immersive reality may provide a ‘better’ and more vivid experience but with increasing computing power and internet bandwidth, it could be tried for important ceremonies. The technology is already being used widely for gaming. The glossary of terms was useful. The student photos were interesting. A photo of a ‘black’ student would have added to the diversity. During the pandemic other institutions could learn from the ‘Bristol’ experience.

Reviewer Expertise:

NA

No decision status is available
